# Association between lymphocytes, hippocampus volume and depressive symptoms in drug – naïve First Episode Psychosis

**DOI:** 10.1192/j.eurpsy.2022.1961

**Published:** 2022-09-01

**Authors:** A. Toll, D. Bergé, K. El-Abidi, T. Legido, V. Pérez-Solà, A. Mané

**Affiliations:** Parc de Salut Mar, Institut De Neuropsiquiatria I Addiccions, Barcelona, Spain

**Keywords:** Lymphocytes, First Episode Psychosis, Hippocampus, schizophrénia

## Abstract

**Introduction:**

The role of the white blood cells, which form the peripheral immune system and are crucial in inflammatory processes, has been laid aside in the context of brain structural changes in schizophrenia.

**Objectives:**

Determine how blood cells are associated with some brain structures volumes in first episode psychosis (FEP) and their relationship with clinical variables at baseline and 1 year follow – up.

**Methods:**

Fifty *drug-naïve* FEP treated between April 2013 and July 2017 at the ETEP Program at Hospital del Mar were included. Inclusion criteria were: 1) age 18-35 years; 2) fulfillment of DSM-IV-TR criteria for brief psychotic disorder, schizophreniform disorder, schizophrenia or unspecified psychosis; 3) no previous history of severe neurological medical conditions or severe traumatic brain injury; 4) presumed IQ level > 80, and 5) no substance abuse or dependence disorders except for cannabis and/or nicotine use. All patients underwent an assessment at baseline and at one-year follow-up, including sociodemographic and clinical variables (substance use, DUP, PANSS, GAF and CDSS). Fasting blood samples were obtained before administering any medication at baseline. Structural T1 MRI was performed at baseline and brain volumes were quantified though FreeSurfer software. SPSS program was used for statistical analyzes.

**Results:**

Lymphocytes have a positive correlation with right and left hippocampus at baseline. Moreover, lymphocytes have a negative correlation with depressive symptoms at baseline and 1 year follow – up.

**Conclusions:**

Lymphocytes may have a protective effect in some brain structures in FEP patients at baseline, especially those implicated in depressive symptoms.

**Disclosure:**

No significant relationships.

